# Elucidating the role of nitrogen plasma composition in the low-temperature self-limiting growth of indium nitride thin films†

**DOI:** 10.1039/d0ra04567e

**Published:** 2020-07-21

**Authors:** Saidjafarzoda Ilhom, Adnan Mohammad, Deepa Shukla, John Grasso, Brian G. Willis, Ali K. Okyay, Necmi Biyikli

**Affiliations:** Department of Electrical & Computer Engineering, University of Connecticut 371 Fairfield Way, Storrs CT 06269 USA necmi.biyikli@uconn.edu +1-860-486-2666; Department of Materials Science & Engineering, University of Connecticut 97 North Eagleville Road, Storrs CT 06269 USA; Department of Chemical and Biomolecular Engineering, University of Connecticut 191 Auditorium Road, Storrs CT 06269 USA; Department of Electrical Engineering, Stanford University Stanford CA 94305 USA

## Abstract

In this work, we have studied the role varying nitrogen plasma compositions play in the low-temperature plasma-assisted growth of indium nitride (InN) thin films. Films were deposited on Si (100) substrates using a plasma-enhanced atomic layer deposition (PE-ALD) reactor featuring a capacitively-coupled hollow-cathode plasma source. Trimethylindium (TMI) and variants of nitrogen plasma (N_2_-only, Ar/N_2_, and Ar/N_2_/H_2_) were used as the metal precursor and nitrogen co-reactant, respectively. *In situ* ellipsometry was employed to observe individual ligand exchange and plasma-assisted ligand removal events in real-time during the growth process. Only the samples grown under hydrogen-free nitrogen plasmas (Ar/N_2_ or N_2_-only) resulted in nearly stoichiometric single-phase crystalline hexagonal InN (h-InN) films at substrate temperatures higher than 200 °C under 100 W rf-plasma power. Varying the plasma gas composition by adding H_2_ led to rather drastic microstructural changes resulting in a cubic phase oxide (c-In_2_O_3_) film. Combining the *in situ* measured growth evolution with *ex situ* materials characterization, we propose a simplified model describing the possible surface reactions/groups during a unit PE-ALD cycle, which depicts the highly efficient oxygen incorporation in the presence of hydrogen radicals. Further structural, chemical, and optical characterization have been carried out on the optimal InN films grown with Ar/N_2_ plasma to extract film properties. Samples grown at lower substrate temperature (160 °C) and reduced/elevated rf-plasma power levels (50/150 W) displayed similar amorphous character, which is attributed to either insufficient surface energy or plasma-induced crystal damage. InN samples grown at 240 °C under 100 W rf-plasma showed clear polycrystalline h-InN layers with ∼20 nm average-sized single crystal domains exhibiting hexagonal symmetry.

## Abstract 2

Hollow-cathode plasma-generated hydrogen radicals induce crystal phase transformation from h-InN to c-In_2_O_3_ during plasma-enhanced atomic layer deposition using trimethyl-indium and Ar/N_2_ plasma.

## Graphical abstract

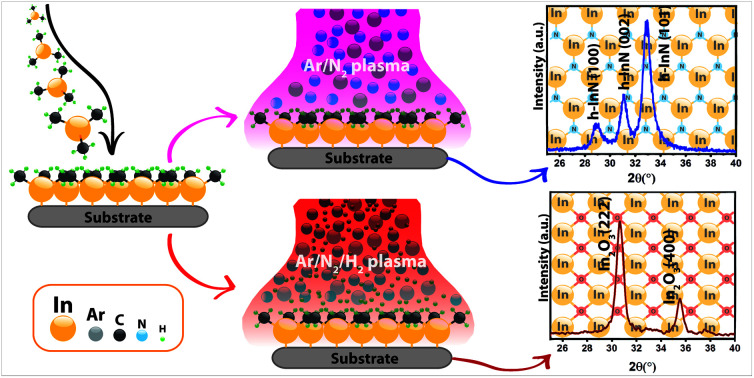

## Introduction

Indium-based III–V compound semiconductors, particularly InN, attract significant interest due to their yet-fully uncovered remarkable optical and electronic properties. High-quality hexagonal crystalline InN has a narrow bandgap (∼0.7 eV) which forms alloys with GaN and AIN (*e.g.* In_1−*x*_Ga_*x*_N) enabling the utilization of a wide spectral range from near-infrared (NIR) to deep-UV wavelengths and therefore offering new opportunities to design LEDs, lasers, and multi-junction solar cells which cover almost the entire terrestrial solar radiation.^1–7^ Additionally, the low electron effective mass of InN leads to a significantly high electron mobility (∼4400 cm^2^ V^−1^ s^−1^) and a high saturation velocity when compared to other mature III–V compound semiconductors such as GaAs and GaN, which positions InN as a unique candidate for high-speed and high-performance electronic devices.^8–12^ However, synthesis of high-quality and defect free InN films *via* conventional growth techniques is quite challenging among other III-nitrides due to its low dissociation temperature, which leads to undesired decomposition into metallic In and N_2_ gas around 500 °C.^13–16^ Conventional synthesis routes such as chemical vapor deposition (CVD), generally utilize NH_3_ as the nitrogen co-reactant, which has relatively poor thermal reactivity and therefore require elevated process temperatures and excessively high (typically >10^4^) NH_3_/TMI (V/III) ratios.^17–20^ Although the use of N_2_ plasma as a co-reactant has been shown to improve nitrogen reactivity in the plasma-assisted metal–organic CVD (PA-MOCVD) producing high-quality crystalline InN films, the process temperatures were still relatively high within the range of 550–775 °C.^21^ Molecular beam epitaxy (MBE) has been another successful route to produce high-quality, single crystal InN films, however MBE also requires similar high substrate temperatures for epitaxial growth.^22–24^ Unfortunately, such harsh process conditions make the realization of InN-based opto-electronic device structures impractical on lower temperature-compatible substrates (CMOS wafers, glass, flexible polymers) and applications (monolithically integrated CMOS/III–V devices, flexible/wearable electronics). Therefore, a significant need and opportunity exists to explore alternative low-temperature material growth techniques towards achieving device-quality InN layers at substantially reduced substrate temperatures.

In this regard, plasma-enhanced atomic layer deposition (PE-ALD) becomes an attractive choice as it offers surface-chemistry driven atomic-scale precision material growth at low substrate temperatures.^17,25–32^ The main advantages of ALD compared to other thin film growth techniques are sub-nanometer precision thickness control, low-temperature growth, ultimate conformality, and large-area uniformity.^33,34^ To date, there have been multiple efforts towards low-temperature InN growth *via* PE-ALD.^17,30–32,35–41^ Initial epitaxial growth of InN at sub-300 °C was achieved by plasma-enhanced atomic layer epitaxy (PE-ALE) where the films exhibited substrate-dependent varying crystallographic orientations.^17,36^ In a relatively recent study, PE-ALD of monocrystalline InN films has also been reported at 250 °C using N_2_-plasma on lower-lattice-mismatched ZnO/Al_2_O_3_ substrates, where the films were fully relaxed with no voids or interlayers at the interfaces.^31^ Moreover, Pedersen *et al.* have recently employed NH_3_-plasma to grow crystalline InN films^38,39^*via* PE-ALD, despite its adverse environmental impacts and corrosivity. Majority of the studies around PE-ALD deposition of InN report on single-phase h-InN films when using hydrogen-free nitrogen plasmas, whereas addition of H_2_ to the plasma gas has resulted in degraded film quality with elevated impurity content and even film porosity.^30–32,35,37^

Although there have been a number of studies on the plasma-assisted ALD of InN employing N_2_/H_2_ and/or N_2_-only plasma compositions, an in-depth investigation of the surface reactions taking place during individual PE-ALD cycles is still significantly missing.^30–41^ In this work, we particularly investigate the role of hydrogen radicals in the surface reactions of TMI with Ar/N_2_ plasma in a custom-designed compact hollow-cathode plasma-ALD reactor. Unlike all previous efforts on PE-ALD of InN utilizing N_2_-plasma, we have observed a major phase transformation from crystalline nitride phase (h-InN) to oxide phase (c-In_2_O_3_) with the addition of H_2_ to Ar/N_2_ plasma gas. To gain further insight into this rather unexpected outcome, we propose probable reaction mechanisms under different plasma chemistries, by corroborating *ex situ* measurements with real-time *in situ* monitored optical film thickness variation data analysis.

## Experimental

Film growth experiments were carried out in OkyayTechALD P100 reactor (Okyay Technologies Inc., Turkey) equipped with a stainless-steel capacitively-coupled hollow-cathode plasma (HCP) source (Meaglow Ltd., Canada). Prior to deposition, Si (100) substrates were cleaned using acetone, isopropanol, and deionized water to remove surface contaminants, and subsequently dried by high-purity N_2_. After loading the samples, the reactor was pumped down to ∼20 mTorr base pressure followed by an *in situ* N_2_-only plasma pre-deposition cleaning at 100 W for 10 min. Trimethylindium (TMI) (Strem Inc., electronic grade, purity ≥ 99.999%) carried by 10 sccm N_2_-flow was used as the metal precursor and variants of nitrogen plasmas (N_2_-only, Ar/N_2_, and Ar/N_2_/H_2_) as co-reactant. 10 s Ar/N_2_ purges at 50/50 sccm were employed each after TMI pulse and plasma exposure to remove the unreacted precursor molecules and surface reaction by-products. Saturation experiments were carried out in the range of 50–200 W rf-plasma power and 30–180 s plasma exposure time, while the substrate temperature varied within 120–240 °C. The saturation curve analyses were carried out by dynamically monitoring the film deposition process in real-time using *in situ* multi-wavelength ellipsometry (MWE) (FS-1, Film Sense, LLC). Initially, film growth is performed until stable growth-per-cycle (GPC) is observed. Then, consecutive 10-cycle runs are done for each TMI pulse time, plasma power, plasma exposure time, and substrate temperature to observe the changes in GPC as well as the thickness changes related to TMI chemisorption and plasma-assisted ligand removal processes, with respect to each parameter set. After completing the saturation curves, thicker (600-cycle) films were grown at substrate temperatures of 160, 200, and 240 °C with a fixed plasma power of 100 W, 30 ms TMI pulse time, 30 s plasma duration, and 10 s purge times for linearity check and further *ex situ* materials characterization.

*Ex situ* optical measurements were done by multi-wavelength ellipsometry (MWE) and a variable-angle spectroscopic ellipsometer (SE) (M-2000 V, J.A. Woollam Co. Inc., NE). MWE unit utilizes 4 visible light emitting diodes (LEDs) as light sources centered at 464.44 nm, 523.56 nm, 599.12 nm, and 637.29 nm, with spectral widths of 28.78 nm, 36.47 nm, 15.17 nm, and 20.54 nm, respectively. The data acquisition time of the MWE component was about 1 s where the ellipsometry angle of incidence was kept constant at ∼70°. Variable-angle SE measurements consist of 390 wavelengths measured across the range of 370–1000 nm simultaneously, with a data acquisition time of 2–5 seconds for the entire spectrum. Data collection is repeated at three angles of incidence (65°, 70°, and 75°) to yield adequate sensitivity across the full spectral range. A model is built and fit to the measured ellipsometry data to extract the thickness and optical constants of target film. The parametric semiconductor (PSEMI) oscillator model is used to describe semiconductor critical points, with precise control of the absorption shape.^42^

Grazing-incidence X-ray diffraction (GIXRD) and X-ray reflectivity (XRR) measurements were performed with Rigaku SmartLab multipurpose X-ray diffractometer (Rigaku Corporation, Japan), operated at 45 kV and 200 mA by using Cu K_α_ radiation. GIXRD measurements were collected at 0.02° step size and 1.0 s counting time in the 2*θ* range of 20°–70°. Omega-2*θ* scan was performed from 0° to 2.0° in 0.01° step size for XRR measurements. Globalfit software was utilized for the reflectivity data fitting process where a three-layer model of InN/SiO_2_/Si has been used. X-ray photoelectron spectroscopy (XPS) with a monochromated Al Kα X-ray source (Kratos AXIS165, U.K.) was utilized for elemental composition, chemical bonding states, and impurity incorporation analysis. Prior to XPS measurements, samples were treated for 10 min in a Novascan UV–ozone cleaner to reduce carbon. FEI Helios Nanolab 460F1 Dual-Beam focused-ion beam scanning electron microscopy (FIB/SEM) (Thermo Fisher Scientific, US) featuring focused Ga-ions was utilized to prepare the samples for high-resolution scanning/transmission electron microscopy (HR-S/TEM) analysis. Prior to FIB operation, the samples were firstly capped by sputter coating of ∼5–10 nm Au–Pd followed by another ∼2 μm Pt coating using the same FIB system to prevent possible InN film crystal damage due to highly energetic Ga ions. The atomic-resolution crystal structure and interface of the InN films were then investigated using cross-sectional TEM imaging *via* FEI Talos F200X system (Thermo Fisher Scientific, US) operated at 200 keV. The same instrument was utilized for HR-S/TEM imaging, which is also equipped with high-resolution energy dispersive X-ray analysis (EDX) unit for elemental mapping.

## Results and discussion

**Fig. 1:**
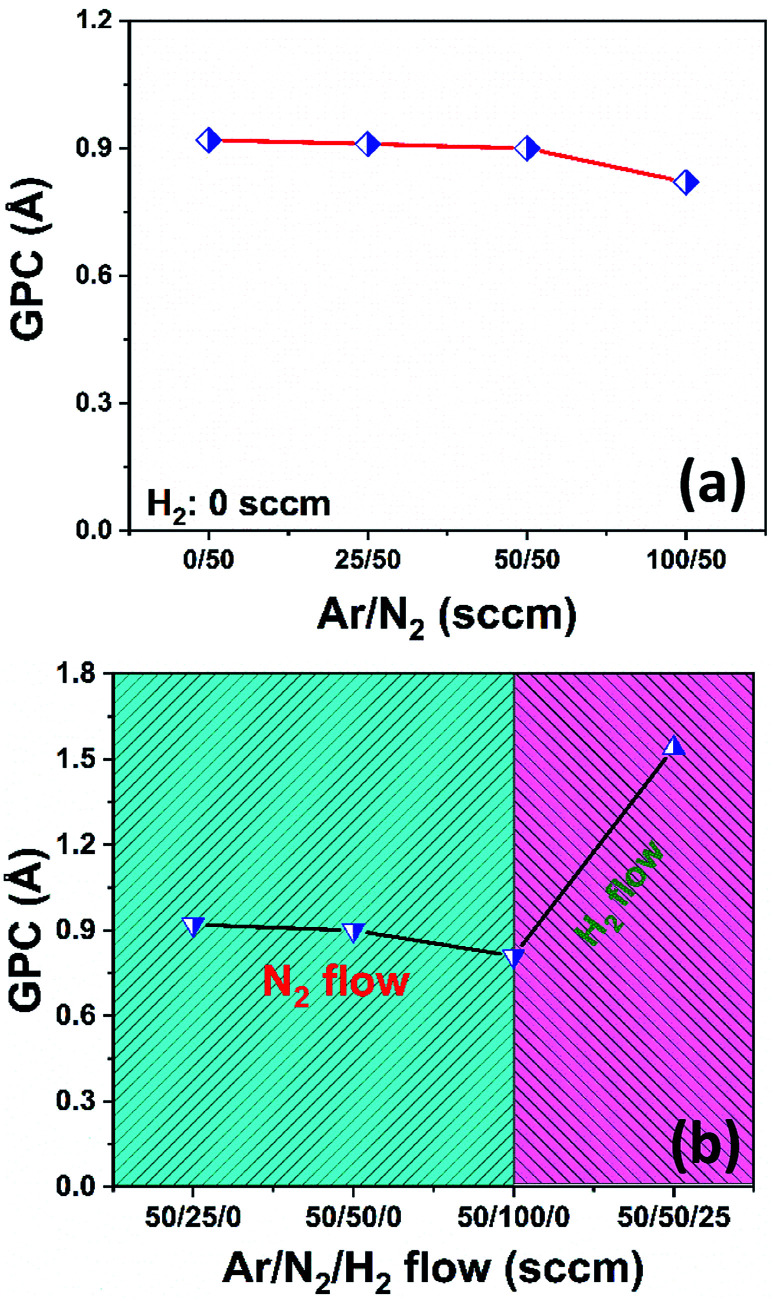
Plasma composition dependence of GPC at 200 °C, 100 W rf-power, 30 s plasma exposure time. (a) Varying Ar-flow while N_2_/H_2_ set at 50/0 sccm. (b) Varying N_2_-flow rates while Ar/H_2_ set at 50/0 sccm, and addition of 25 sccm H_2_ while Ar/N_2_ set at 50/50 sccm.

**Fig. 2:**
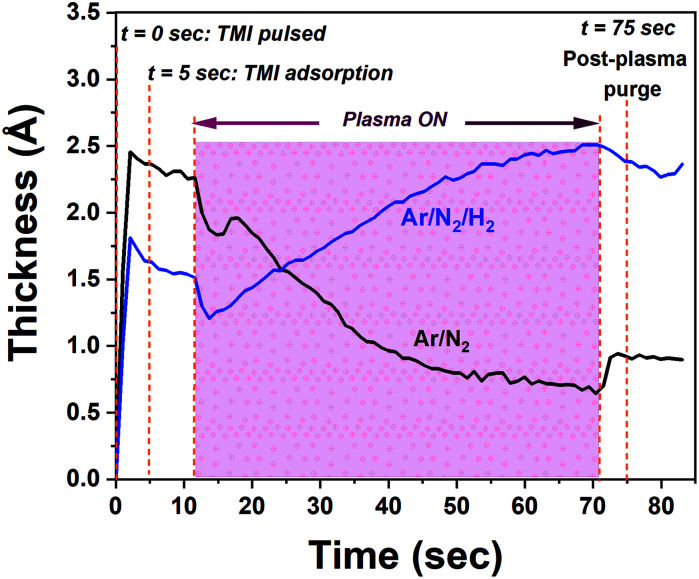
Averaged *in situ* unit ALD-cycles for the film growth using Ar/N_2_ (50/50 sccm) and Ar/N_2_/H_2_ (50/50/50 sccm) plasma compositions, at 100 W rf-plasma power, for 60 seconds exposure and 200 °C substrate temperature. 0, 5, and 75 s represent selected instances of TMI pulsing, TMI being adsorbed onto the substrate surface while chamber purging continues, and post-plasma exposure purge period, respectively.

We initially studied the effect of varying plasma chemistries on InN film deposition at 100 W rf-power and 200 °C substrate temperature. Fig. 1 shows the measured growth-per-cycle (GPC) parameter as a function of changing plasma gas composition. The amount of film thickness gain remained nearly constant in the range of 0–100 sccm Ar-flow with N_2_/H_2_ set to 50/0 sccm (Fig. 1a). Similarly, varying the N_2_ gas flow in the range of 25–100 sccm while keeping Ar/H_2_ at 50/0 sccm resulted in a relatively constant GPC as well (Fig. 1b). However, adding 25 sccm of H_2_ into the Ar/N_2_ plasma composition, led to a rather drastic increase in the recorded GPC (Fig. 1b). At first glance, this increase in the film deposition rate signals a possibly more efficient removal process of the –CH_3_-ligand groups from the surface due to the presence of highly energetic hydrogen radicals in the plasma gas mixture, and thus increasing the film deposition rate by enhancing the efficiency of nitrogen incorporation. To gain a better understanding of the impact hydrogen radicals have on the surface reactions of TMI with Ar/N_2_ plasma, we have carried out consecutive 10-cycle runs using Ar/N_2_-only and Ar/N_2_/H_2_ plasma mixtures, while dynamically monitoring the film thickness variation in real-time *via in situ* ellipsometry. Fig. 2 shows how the film thickness during a single ALD cycle is affected by the addition of H_2_ into the Ar/N_2_ plasma gas. Analyzing the plasma exposure period for the Ar/N_2_ mixture in Fig. 2, except for rf-power induced artifacts (kinks) at around 20 seconds and after the plasma is turned off, the rather sharp decay clearly indicates the effective Ar/N_2_ plasma-assisted removal of large methyl (–CH_3_) ligand groups. However, examining the plasma half-cycle trend for the Ar/N_2_/H_2_ plasma composition in Fig. 2, there appears to be a rather continuous thickness gain during the entire plasma exposure, which is not typical for PE-ALD reactions.

**Fig. 3:**
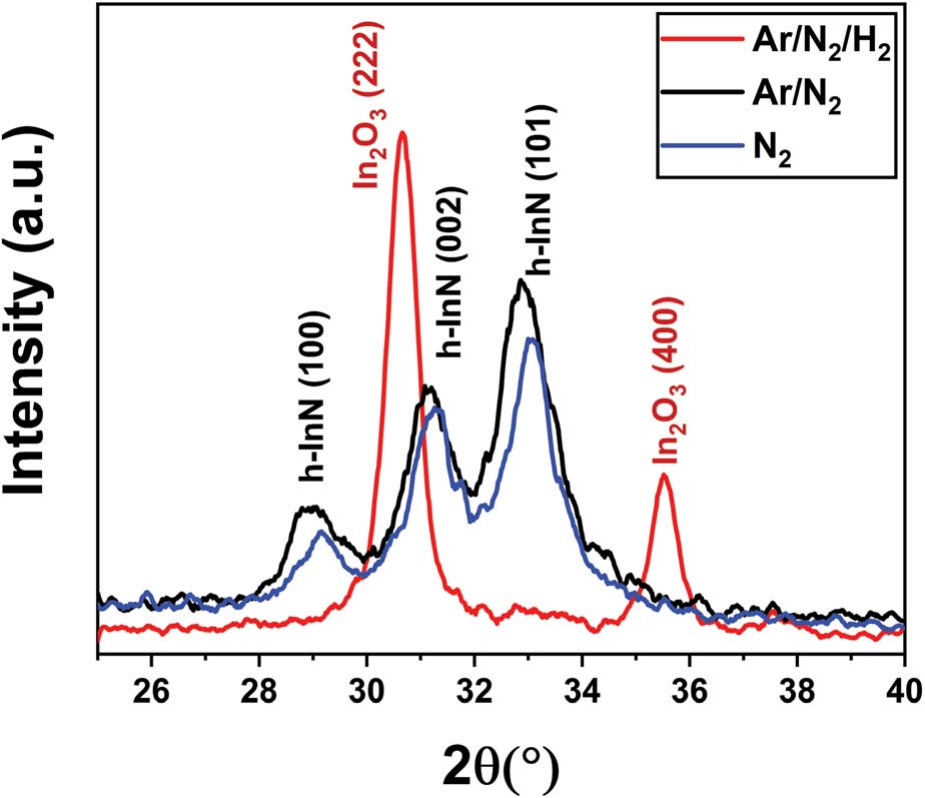
GIXRD measurement for the film growth using Ar/N_2_/H_2_ (50/50/50 sccm) Ar/N_2_ (50/50 sccm), and N_2_ (50 sccm) plasma compositions, 100 W rf-plasma power and 200 °C substrate temperature depicting the completely different crystalline phase obtained with the addition of hydrogen into the plasma gas.

To gain further insight into the possible effects of varying the plasma chemistry on the crystalline properties of the deposited films, we have carried out grazing-incidence X-ray diffraction (GIXRD) measurements, as depicted in Fig. 3. The acquired GIXRD data revealed that the samples grown under Ar/N_2_ and N_2_-only plasmas exhibit hexagonal InN crystal structure with (100), (002), and (101) wurtzite phase reflections between 2*θ* = 25°–40° scan range. On the other hand, when H_2_ is added to the plasma gas flow, Ar/N_2_/H_2_ plasma leads to a substantial microstructural transformation, forming an oxide phase instead of nitride, *i.e.*, crystalline In_2_O_3_ film with relatively strong (222) and (400) cubic-phase crystal orientations (Fig. 3). The possible reasons for this phase transformation including probable reaction mechanisms and forming surface groups will be further elaborated in the following sections.

**Fig. 4:**
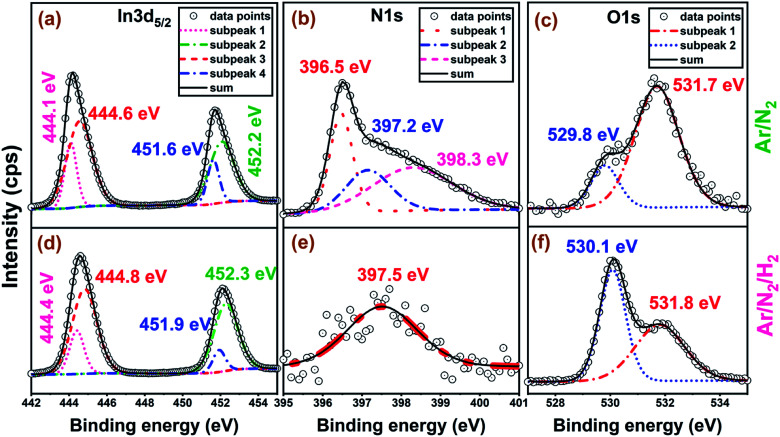
High-resolution XPS measurements of the 300-cycle films grown at 200 °C, 100 W (a–c) with Ar/N_2_ and (d–f) Ar/N_2_/H_2_ plasma chemistries.

XPS was used to quantify film stoichiometry and elemental composition, along with the relevant chemical bonding structure for different plasma chemistries utilized. High-resolution XPS scans for Ar/N_2_ and Ar/N_2_/H_2_ samples are shown in Fig. 4, while the elemental composition data are summarized in Table 1, where the quantification is based on the full integrated area of the N 1s and In 3d regions. The scans are shown for as-grown air-exposed samples without any Ar-sputtering mainly because preferential sputtering was observed for the lighter elements, which makes elemental analysis of InN quite a challenging task even with highly surface-sensitive techniques like XPS.^35,43–46^ XPS analyses of nitride films featuring Ar-sputtering processes typically results preferential etching of the lighter element, nitrogen, leading to the formation of metallic indium bond formation, significantly hindering the measurement of the actual bulk film stoichiometry. Although measuring the film stoichiometry and impurity content from the un-sputtered samples might lead to the overestimation of species closer to the surface (such as atmospheric C and O contaminations), the In to N ratios would not be strongly affected by an overlayer and could be rather considered correct in their relative stoichiometry estimations.^23^ Hence, the relatively high carbon and oxygen concentrations on the sample surface are consistent with prior ALD studies of InN materials.^23,35,38,44,47,48^ In addition, we have performed a UV–ozone surface treatment process immediately prior to loading the samples into the XPS chamber, which helped reducing the surface carbon content while slightly increasing surface oxygen concentration. While the films grown with hydrogen-free plasmas (Ar/N_2_ and N_2_-only plasma) exhibit nearly ideal In : N stoichiometry values, a significant increase is observed (>5 : 1) in the samples deposited using Ar/N_2_/H_2_ plasma (Table 1). This rather drastic change is mainly reflected due to the much lower N incorporation in the films (∼5.0 at%), while the relative O concentration shows a sharp increase to ∼44.0 at%. On the other hand, the In and C compositions stay relatively similar to Ar/N_2_ and N_2_-only plasma samples (Table 1). The In : O stoichiometry for Ar/N_2_/H_2_-plasma grown film is calculated to be close to (2 : 3), which confirms the crystalline c-In_2_O_3_ phase formation in this sample.

**Table 1:** Chemical composition of the 300-cycle InN samples for varying plasma gas composition in terms of atomic concentration (measured from the surface of the films)

Ar/N_2_/H_2_ (sccm)	In at%	N at%	O at%	C at%
50/50/0	28.2	29.7	17.9	24.2
0/50/0	24.4	27.6	18.7	29.3
50/50/50	26.4	5.0	44.0	24.0

The O 1s region is well-described by two major components with Ar/N_2_ samples having binding energies (BEs) at 529.8 eV and 531.7 eV. The upper and lower binding energy components are assigned to surface and bulk-like oxygen components, respectively. These assignments are based on prior literature as well an oxidized indium foil reference, where the upper binding energy component is removed after light sputtering, and the lower binding energy component remains in the bulk.^23,44,47–51^ The assignments are consistent with the data in Fig. 4c and f where the higher O content films have relatively more of the lower binding energy component, and the higher N content films have mostly the higher binding energy surface component.

The N 1s region for Ar/N_2_-plasma sample shows a sharp feature at 396.5 eV, and a broad region to higher binding energy. Some previous works on ALD deposited InN films have fit the N 1s region with only two components, but here the narrow peak for the low binding energy component necessitates at least three components for a quality fit.^35,38^ The lower BE peak at 396.5 eV is assigned to nitrogen in the form of InN.^23,31,32,35–41,43–53^ For Ar/N_2_-plasma samples, the additional components are at 397.2 and 398.3 eV. The upper component is preserved after sputtering into the bulk of the film and can be assigned to an In–N–O bonding environment, which leaves the middle component unassigned. Previous studies of InN films deposited by molecular beam epitaxy (MBE) assigned a component at 397.7 eV to excess N in the film, but others have observed a NO_x_ component at 397.5 eV from air exposure, which seems most likely be the case for the present films.^49,54^ Also, the almost faded N 1s peak to a weak signal at 397.5 eV and elevated bulk-type O 1s signal at 530.1 eV for Ar/N_2_/H_2_ sample (Fig. 4e and f), further supports our claim of the phase transformation from hexagonal nitride (InN) to cubic oxide (In_2_O_3_) structure as evidenced by GIXRD under hydrogen-containing plasmas.

The In 3d peak has two major components with In 3d_5/2_ binding energies of 444.1 and 444.6 eV, where the lower binding energy component is rather narrow and the upper is broad. In the literature, the In 3d_5/2_ peak is conventionally deconvoluted to metallic indium (In–In), In–N, and In–O bonds, where the relative shifts between these bonding states have been used in assigning individual sub-peaks.^45^ Majority of the prior works have reported In–O peaks at ∼1 eV higher in binding energy compared to In–N, where In–N is located at about 0.3–0.5 eV higher than the metallic indium sub-peak assigned at ∼443.5–443.8 eV.^23,37,39,53–56^ Our data are similar to other XPS studies of ALD deposited InN films.^38^ Some previous studies have interpreted these upper and lower binding energy peaks (Fig. 4a) as In–O and In–N bonding contributions, respectively, but that assignment is problematic for several reasons. Firstly, the appearance of two components has no correlation with the nitrogen content of the films. The two components are present for both stoichiometric films with In : N ratios of 1 : 1 and films with substantially reduced N content (Fig. 4a and d). In fact, similar In 3d components were observed for reference indium oxide thin films. Previous studies of indium oxide thin films have offered at least two explanations for the two components. The first is that the two components are different final states from a screening mechanism that involves the excitation of plasmons.^57^ In the previous studies, the In 3d peak was characterized by an asymmetric line shape with two overlapping components with a separation near 0.5 eV, where the high binding energy component is more intense, is broader, and has more Lorentzian character.^58^ All of these features are also seen here for these ALD deposited InO_*x*_N_*y*_ films: the higher binding energy peak is broader and more intense and has more Lorentzian character. In the prior studies, evidence for the plasmon has been weak, and alternative explanations include attributing the asymmetry to oxygen vacancies.^59^ The latter is not directly translatable to these ALD films, and we have no evidence for analogous nitrogen vacancies. In either case, we cannot reliably attribute the two In 3d components with In–N and In–O bonding. However, N 1s and O 1s high-resolution spectra enable us to draw a more decisive conclusion with regards to h-InN to c-In_2_O_3_ phase transformation.

**Fig. 5:**
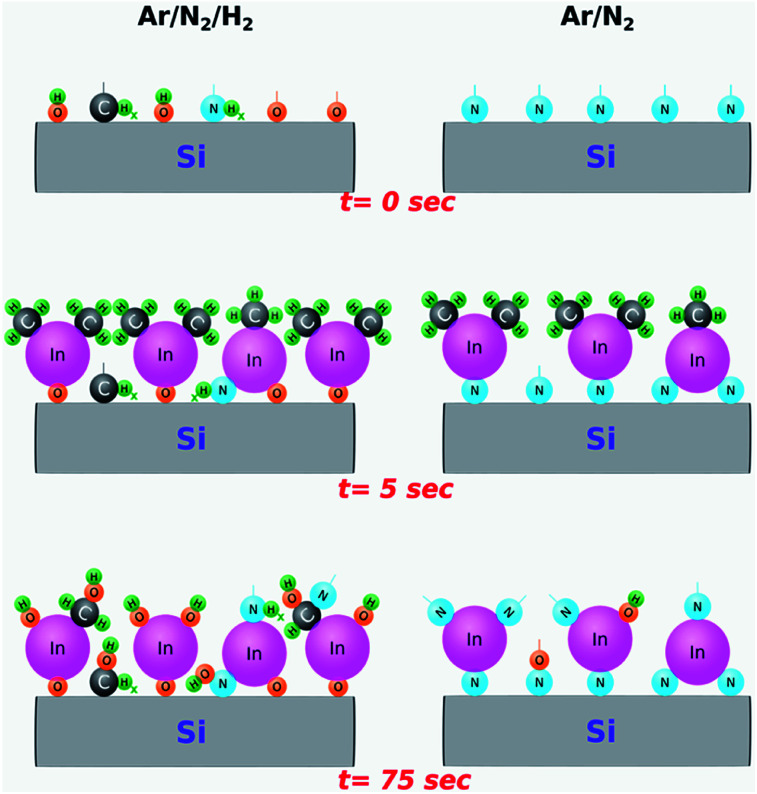
Graphical representation of the proposed surface reactions and the resulting surface groups during a unit ALD cycle at the selected instances during a unit PE-ALD cycle with Ar/N_2_ and Ar/N_2_/H_2_ plasma chemistries. 0, 5, and 75 s represent selected instances of TMI pulsing, TMI being adsorbed onto the substrate surface while chamber purging continues, and post-plasma exposure purge period, respectively.

Although the exact plasma–surface interaction mechanisms and reaction pathways taking place in a PE-ALD environment are rather complex and challenging to model, based on our real-time *in situ* observations and *ex situ* measurement results, we attempt to provide an explanation for the rather strong influence of hydrogen radicals. Fig. 5 represents a simplified cartoon-based model, depicting the formation of possible surface groups on an already exposed surface to the respective plasma chemistries (Ar/N_2_/H_2_ or Ar/N_2_). The instances selected correspond to right before TMI pulsing (0 s), In precursor molecules adsorbed to pre-occupied surface groups (5 s), and after the plasma half-cycle (75 s), with the reactor chamber being purged and ready for the next TMI pulse.

With this simplified model, we hypothesize the following two surface reaction scenarios as a function of plasma chemistry: (i) at *t* = 0 s, just before the TMI pulsing, the sample surface exposed to Ar/N_2_ plasma is possibly enriched mostly with nitrogen groups due to high radical flux density produced by the large-diameter hollow-cathode plasma source. This, in turn, promotes efficient In–N binding during plasma half-cycles leading to the h-InN film structure, as evidenced by GIXRD data (Fig. 3). (ii) On the other hand, adding H_2_ to Ar/N_2_ plasma might lead to a surface terminated with a combination of –CH_*x*_, –NH_*x*_, and HCN^−^ surface groups, possibly due to the reaction by-products being decomposed in the plasma and re-deposited (plasma re-deposition) on the sample surface.^60,61^ In addition, hydroxyl (–OH) terminated groups originating partially from the residual H_2_O molecules within the chamber,^60,61^ might also contribute to the continuous thickness gain during the Ar/N_2_/H_2_ plasma exposure, which will result in the efficient formation of In–O surface groups (Fig. 2). Comparing these possible reaction mechanisms, we believe that the contribution from the plasma re-deposited species (–CH_*x*_, –NH_*x*_, and HCN^−^) should be negligible at the plasma conditions used in this experiment (100 W for 30 seconds), which is supported by the XPS analyses that showed no detectable carbon content after mild Ar-sputtering (not shown). As such, we believe that hydrogen radicals within the plasma environment mainly lead to the formation of hydroxyl (–OH) surface groups. In fact, it has been shown that when H gets involved during InN film growth, it has a strong tendency towards binding to N, which leads to breaking or weakening the In–N chemical bond.^62^ Therefore, given the low thermal stability of In–N bonding structure^13–16^ combined with the highly energetic plasma-induced hydrogen radicals, the probability of In–N chemical bond dissociation would increase, leaving vacated indium sites to be easily bonded with the residual water vapor and oxygen to form hydroxyl (–OH) terminated surfaces. Consequently, during the subsequent In precursor pulsing half-cycle, TMI molecules are readily adsorbed to the –OH groups and lead to the growth of crystalline In_2_O_3_ layers. We would like to point out that this effect is most probably related to a unique chemical response of elemental In or in its precursor form (TMI) to the hydrogen radicals, because very recently under almost identical experimental conditions using the same ALD system we had successfully grown high-quality and near-ideal stoichiometric AlN, which showed <5% oxygen and no detectable carbon in the bulk of the films with trimethyl-aluminum and the very same Ar/N_2_/H_2_ plasma chemistry.^33,34^ Hence, the exact reasons as to why and how the hydrogen radical content in the Ar/N_2_/H_2_ plasma leads to more efficient O incorporation in InN growth but not in AlN, as well as how Ar/N_2_-only plasma prevents this in InN still remains largely unknown at this point and needs further experimental investigation backed up with theoretical calculations which can simulate the impact of different plasma species.

To summarize our developed understanding based on this proposed model, we can extract the following main outcomes: (i) the self-limiting and near-ideal stoichiometric film growth of h-InN is achieved with both Ar/N_2_ and N_2_-only hydrogen-free plasma compositions. (ii) Addition of hydrogen to the Ar/N_2_ plasma chemistry entirely changes the growth behavior and leads to crystalline oxide films (c-In_2_O_3_), a completely different film phase and structure. Thus, in an attempt to better investigate the film properties of the targeted h-InN layers, we have performed further growth and characterization experiments under Ar/N_2_ plasma in the remaining sections of the manuscript, unless stated otherwise.

**Fig. 6:**
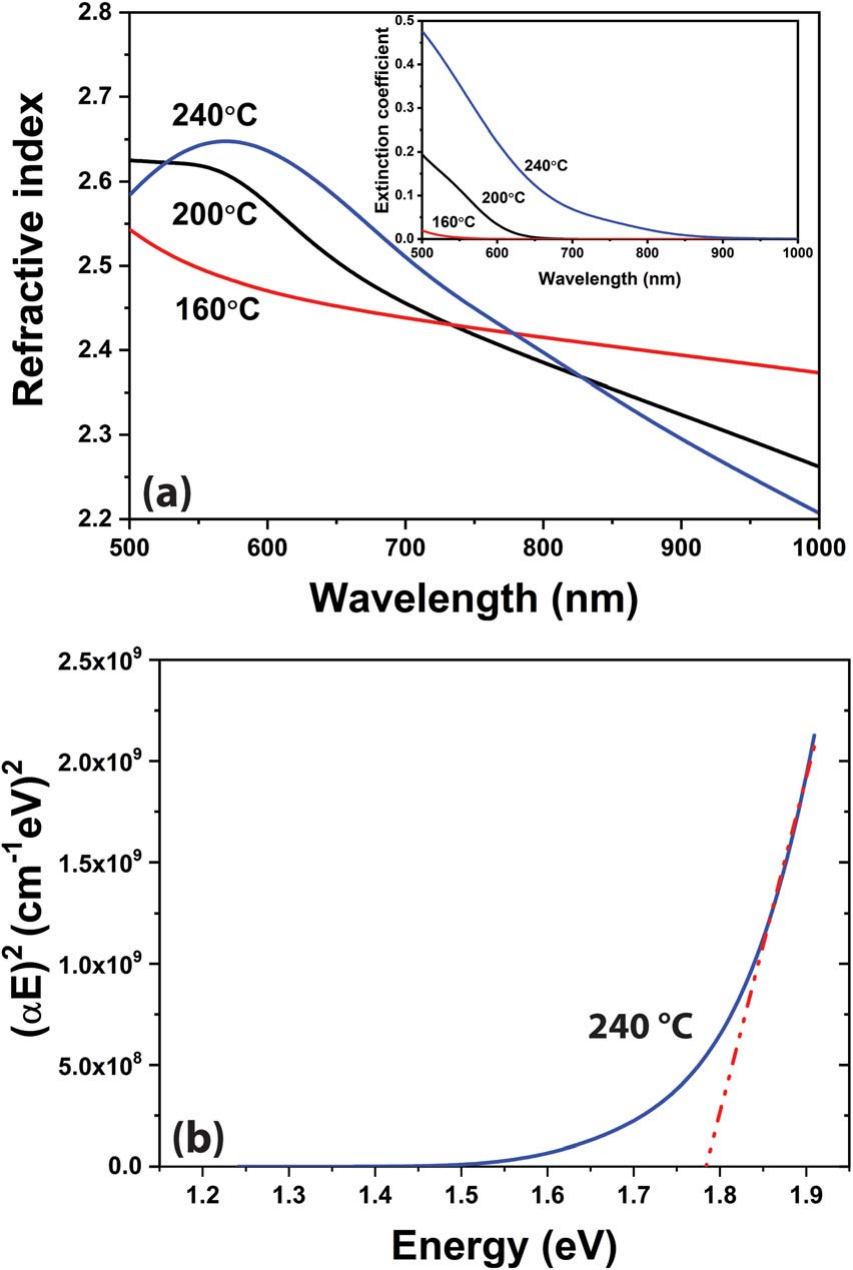
(a) *Ex situ* spectroscopic ellipsometer measurements of the spectral refractive index for the 600-cycle InN films grown at varying substrate temperatures and 100 W rf-plasma power using Ar/N_2_ plasma composition. (Inset) Extracted spectral extinction coefficient for the same samples. (b) Spectral absorption behavior of InN film deposited at 240 °C, along with the extracted absorption band edge fit.

Film growth saturation studies for Ar/N_2_-plasma samples are summarized in Fig. S1,† where average GPC values are extracted from *in situ* ellipsometry recorded thickness changes. The GPC values showed a relatively constant trend with respect to TMI pulse time, purge period, and plasma duration; while, plasma power exhibited a rather non-saturating, increasing GPC behavior with elevated rf-power, as shown in Fig. S1.† However, GIXRD analysis of the films grown at optimized conditions depicted that only 100 W rf-power enabled crystalline h-InN growth; whereas both lower and higher rf-power usage (*e.g.* 50 and 150 W) resulted in films with amorphous signature (Fig. S2†). The reason for the amorphous film character at lower plasma power can be attributed to the insufficient energy of plasma species to facilitate crystallization,^34^ while at higher rf-power either plasma-induced crystal damage or ligand re-deposition might have become dominant, both resulting in a degraded crystal structure.^60,61^ Whereas our multi-wavelength ellipsometer (MWE) proved to be very useful during real-time process monitoring, *ex situ* spectroscopic ellipsometer (SE) enables additional critical insight into the material dispersion or change in index of refraction across a broad wavelength spectrum. Examination of Fig. 6a reveals the refractive index of the films grown at 200 and 240 °C to exhibit anomalous dispersion behavior due to the presence of considerable absorption characteristics in the wavelength range, as seen in the inset of Fig. 6a. The film grown at 160 °C behaves normally but deviates from the other films suggesting a difference in either composition or crystal structure. XRD analysis will reveal this film to be amorphous, while the other (200 and 240 °C) films are crystalline, which confirms this variation. Table 2 illustrates the differences in the capabilities of the MWE and variable angle SE techniques. Good agreement is shown for the film grown at 160 °C; however, noteworthy deviations appear for the films grown at higher substrate temperatures. The 160 °C sample is nearly transparent and can be accurately characterized by the MWE, whereas the increasing absorption character of the films grown at higher temperatures is more accurately characterized by the variable-angle SE. The measured SE thickness of the film grown at 240 °C (45.6 nm), is in good agreement with the thickness extracted from the XRR analysis (∼44.2 nm) and HR-TEM images (44.0 nm).

**Table 2:** Film thickness, GPC, and refractive index measurement results obtained for 600-cycle HCPA-ALD grown InN samples at 30 s, 100 W rf-power and different substrate temperatures *via ex situ* multi-wavelength and spectroscopic ellipsometry

*T*_sub_ (°C)	*Ex situ* MWE			*Ex situ* SE		
	*t*_avg_ (nm)	GPC (Å)	*n*_avg_	*t*_avg_ (nm)	GPC (Å)	*n*_avg_
160	93.34	1.56	1.98	96.33	1.61	1.98
200	63.58	1.06	2.40	73.50	1.23	2.53
240	38.05	0.63	2.62	45.65	0.76	2.62

The absorption coefficient,1
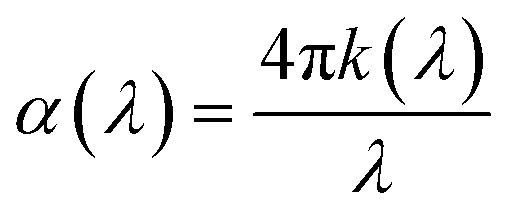
is extracted from the extinction coefficient, *k*(*λ*), calculated from the SE data. The optical band gap (absorption band edge), *E*_G_, is then estimated for InN, a direct band gap material, with the following expression:

2 *αE* = *A*(*E* − *E*_G_)^1/2^

The band gap is obtained *via* linear extrapolation corresponding to the onset of the absorption spectrum to (*αE*)^2^ = 0.^35^ The Tauc plot in Fig. 6b demonstrates the method to estimate the band gap at 240 °C. The band gaps for 200 and 160 °C are extracted in similar manners, in Fig. S3,† estimated at 2.10 eV and 2.24 eV, respectively. As the growth temperature increases, the band gap decreases; at 240 °C the estimated band gap is 1.78 eV which agrees well with previously ALD-grown InN films.^35,38^ The differences in the optical band gap values could be attributed to the different structural properties of the grown films: it has been shown that various factors can influence the band gap energy, *i.e.* lattice parameter, impurities, lattice strain, crystallite size, *etc.*^63^ We can safely claim that the sample with the highest structural quality (240 °C) shows the smallest absorption band edge, which is indicative of lower impurity concentration and crystal imperfections while reminding larger crystallite sizes. However, the amorphous sample (@ 160 °C) exhibits the largest optical band gap with reduced band-to-band absorption, mainly due to elevated impurity incorporation. In fact, this can be further confirmed by analysing the GPC and refractive index (@ 633 nm) trend of the films as a function of the substrate temperature. The GPC decreases from 1.61 to 0.63 Å and the refractive index increases from 1.98 to 2.62 when the substrate temperature is increased from 160 to 240 °C, respectively (Table 2). The drop in GPC can be attributed to the more efficient removal of bulky (–CH_3_) ligand groups, where the elevated refractive index reminds of possible densification and thus enhanced crystallization of the films at higher (240 °C) substrate temperature.

**Fig. 7:**
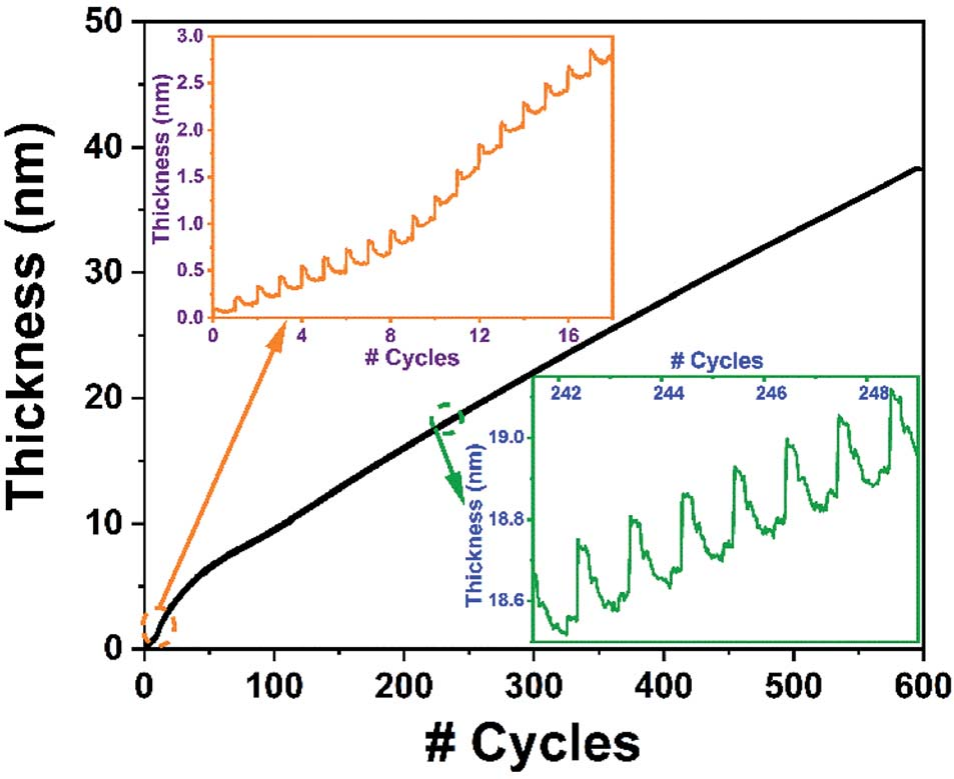
*In situ* ellipsometry recording of the 600-cycle InN film growth experiment at 240 °C using 100 W rf-power using Ar/N_2_ plasma composition. (Insets) Zoomed-in ALD cycles showing the individual TMI chemisorption and plasma-assisted ligand exchange/removal events.

Fig. 7 shows the real-time *in situ* recorded data of 600-cycles sample grown at 240 °C using 100 W rf-power. Mainly, the film exhibits two different modes of growth, with a rather sharp GPC at the early stages Fig. 7 (inset 1), followed by a steady-state linear growth after about the 50th cycle. Such a high GPC gain at the initial stage of deposition at elevated rf-powers has been primarily attributed to a plasma-induced substrate-enhanced nucleation onset, which later stabilizes at lower values in the remaining linear regime.^34,64,65^Fig. 7 (inset 2) shows the magnified ALD-cycles depicting the individual TMI chemisorption and plasma-assisted ligand removal events over the linear region of the film growth.

**Fig. 8:**
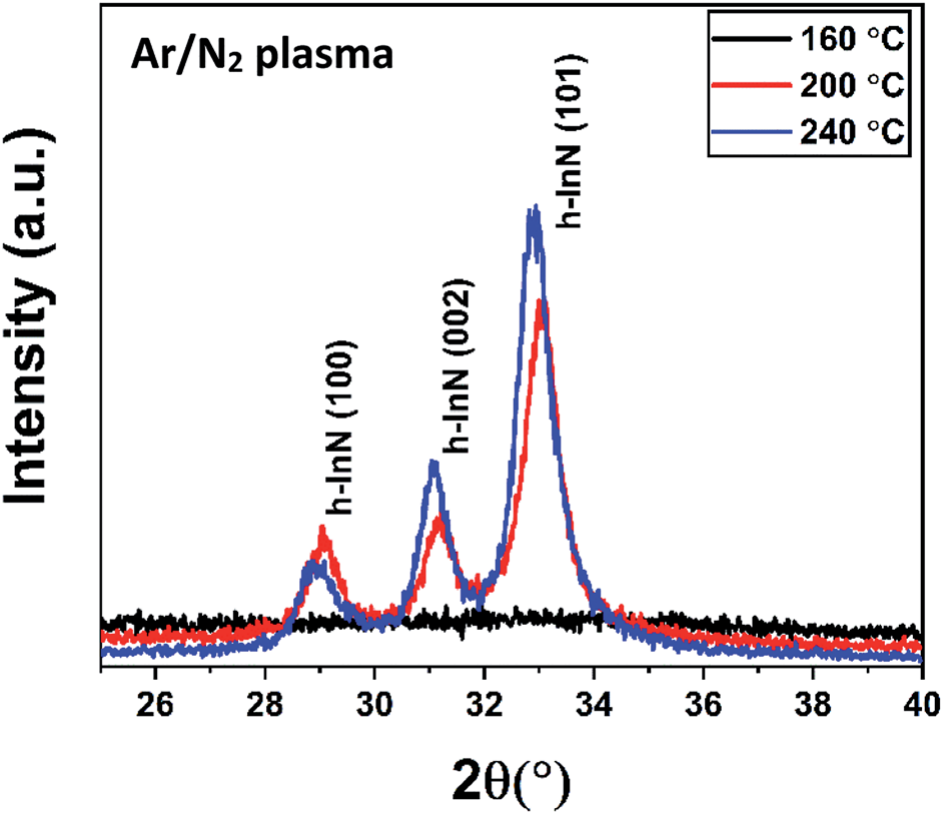
GIXRD measurement for 600-cycle InN samples grown at variable substrate temperatures with 100 W rf-power for 30 s plasma exposure time.

The structural properties of samples grown under varying substrate temperatures were studied using GIXRD, as shown in Fig. 8. The GIXRD measurement revealed mainly the (100), (002), and (101) reflection peaks of the hexagonal wurtzite (h-InN) crystal structure for the films grown at 200 and 240 °C, while the 160 °C sample exhibited amorphous character, possibly due to inadequate thermal energy component required to facilitate crystallization at this temperature. The preferred crystal orientation for the films appears to be (101), where the relatively higher peak intensity at 240 °C signals better crystal quality films compared to 200 °C. Furthermore, a slight left shift of peak reflections at 240 °C reminds of possible thermal induced stress build-up within the bulk of InN film. Using the full-width-at-half-maxima (FWHM) obtained from the GIXRD data, the average crystal grain size values were calculated by Scherrer equation:3
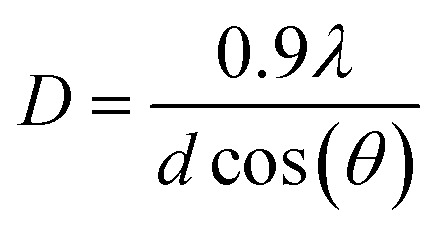
where *D* is crystallite size, *λ* is the X-ray wavelength, *d* is the FWHM of the XRD peak of interest, and *θ* is the angle in radians. The calculated grain sizes for (101) reflections are summarized in Table 3. The grain size of the films increased slightly from 19.3 nm at 200 °C to 19.8 nm at 240 °C, signalling a rather incremental crystal structure improvement, which is also evidenced by the narrowing of FWHM and the GIXRD peak intensity increase (Fig. 8) at this temperature.

**Table 3:** Structural material parameters extracted from GIXRD and XRR measurements of the 600-cycle InN samples grown at 30 s, 100 W rf-power and different substrate temperatures

*T*_sub_ (°C)	GIXRD			XRR		
	(101) position (°)	FWHM (°)	Grain size (nm)	Density (g cm^−3^)	Thickness (nm)	Roughness (nm)
160	—	—	—	4.26	93.52	1.15
200	33.06	0.75	19.28	5.20	65.03	1.81
240	32.93	0.73	19.81	6.26	44.21	2.13

**Fig. 9:**
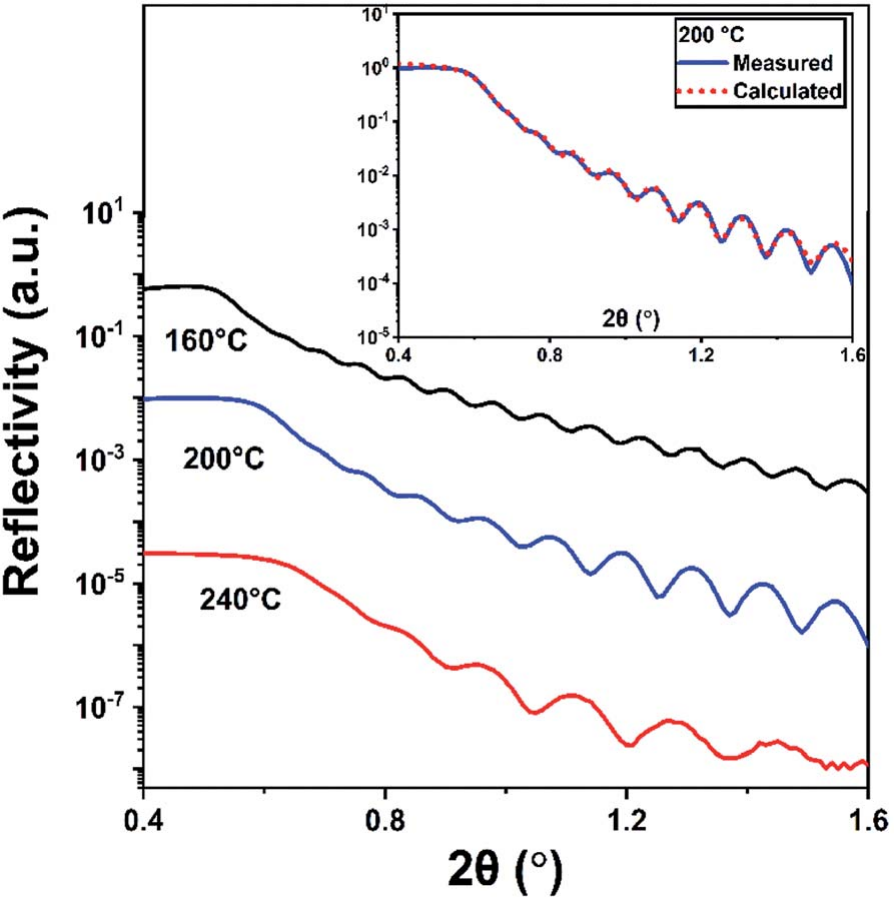
XRR measurements of the four 600-cycle PEALD grown InN thin film samples using Ar/N_2_ plasma composition. (Inset) The measurement and software-fitted calculation data for the samples grown at 200 °C.

The density, thickness, and surface roughness of InN films grown at varying substrate temperatures were studied using X-ray reflectivity (XRR) technique. Fig. 9 shows the measured XRR curves with the inset graph depicting the data for 200 °C fitted with the model calculation, where each measurement curve was manually offset along the *y*-axis for better clarity. The extracted data from the analysis is summarized in Table 3. The density of the films significantly increases from 4.26 to 6.26 g cm^−3^ with the increase in substrate temperature from 160 to 240 °C confirming the densification and therefore crystallization of the InN films, which are in good agreement with previously reported values.^35,38,66^ In fact, XRR reveals that there is significant crystallinity enhancement from 200 to 240 °C, as inferred from the comparable difference between their respective densities being at 5.20 and 6.26 g cm^−3^, Table 3. It should be noted that this significant improvement falls undetected in GIXRD, which shows the film crystallite sizes at both of these temperatures do not vary considerably, although there is a slight change in their GIXRD peak values (Table 3 and Fig. 8). Additionally, this is reflected in the surface roughness of the samples, which exhibits an increase from ∼1.2 nm at 160 °C to about 2.1 nm at 240 °C possibly due to the formation of crystal grains adding up to the surface roughness. The XRR-extracted thickness values ranged between 44.2–93.5 nm which are also in good agreement with the thickness values measured *via* ellipsometry (Table 2).

Table 4 summarizes the XPS extracted elemental composition of the InN films grown using Ar/N_2_ plasma with respect to substrate temperature, which are measured from the surface of the samples after UV–ozone treatment to reduce possible surface carbon contaminants due to post-deposition atmospheric exposure. Although the atomic concentration of O and C does not diminish significantly as measured from the surface, comparing their relative compositions between all sample sets provides reasonable insight about possible stoichiometry within the bulk of the films. While the O content stays fairly the same at about 19.8–19.6 at% for the samples grown at 200 and 240 °C, the C impurity goes down from 26.3 at% to about 20.9 at% at 240 °C, possibly relating to a more efficient methyl (–CH_3_) ligand removal process at higher substrate temperatures. Furthermore, at 240 °C an indium-rich film composition is observed with an In/N ratio of ∼1.3; whereas, at 200 °C the samples become slightly nitrogen-rich with an In/N ratio of ∼0.9. It should be noted that the slight stoichiometric discrepancy between the Ar/N_2_-plasma grown films at 200 °C (in Tables 1 and 4), which are for two different samples (300 *vs.* 600-cycle) might be due to variations in the sample surface preparation (UV–ozone cleaning after atmospheric exposure) and measurement conditions. On the other hand, the sample grown at 160 °C shows significantly reduced In content at 14.5 at% with relatively high O and C impurity levels at 21.2 and 35.2 at%. This conforms well with the results of amorphous film characteristics revealed by GIXRD (Fig. 8), as well as elevated GPC together with low refractive index obtained *via* MWE and SE (Table 2), indicative of a possibly porous film structure with high impurity content.^35^

**Table 4:** Chemical composition of the 600-cycle InN samples as a function of substrate temperature in terms of atomic concentration (measured from the surface of the films)

*T*_sub_ (°C)	In at%	N at%	O at%	C at%
160	14.5	21.9	21.2	35.2
200	25.0	28.9	19.8	26.3
240	33.6	25.9	19.6	20.9

**Fig. 10:**
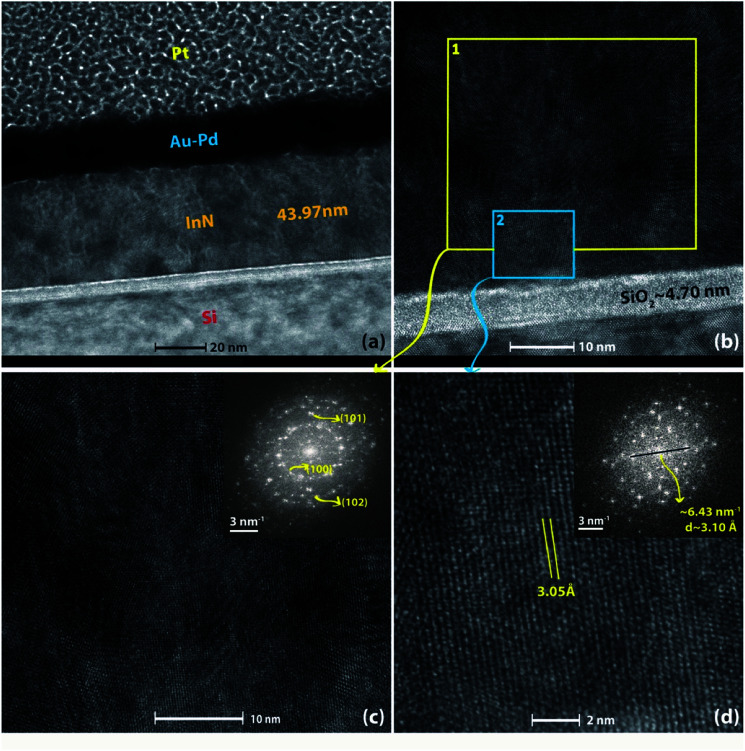
(a) HR-TEM image of 600-cycle InN film deposited at 240 °C with 100 W rf-power using Ar/N_2_ plasma composition. (b) HR-TEM image showing InN film layer and the interface with Si substrate. (c) Zoomed-in micrograph showing high-resolution lattice features of InN film selected over region (1), (inset) Fast Fourier Transform (FFT) of the film crystal structure under the selected-region (1). (d) Zoomed-in micrograph showing high-resolution lattice features of InN film selected over a smaller area, region (2), (inset) Fast Fourier Transform (FFT) of this single-crystal-like film structure which depicts the hexagonal crystal symmetry rather than the polycrystalline rings.

High-resolution TEM analysis was performed on InN film grown at 240 °C to study the crystal structure and interfacial layer more in detail. Fig. 10 shows the cross-sectional TEM image where the thickness of the sample was found to be ∼44 nm, which is in good agreement with the thickness measurements obtained using SE (Table 2) and XRR (Table 3). Fig. 10b depicts the interface of InN to Si substrate with about 4.70 nm amorphous native oxide (SiO_2_) layer. Fig. 10c and d are zoomed-in HR-TEM micrographs over a larger region (#1) and another over a smaller region (#2) as depicted on Fig. 10b, which reveal the polycrystalline nature of InN film where several individual crystal domains with different crystallographic orientations are clearly observable. Additionally, to gain further insight about the possible crystal orientations, fast fourier transform (FFT) analysis was performed on both of these micrographs shown as insets to Fig. 10c and d. FFT over the broader region (#1) clearly shows scattered arc-like diffraction rings corresponding to (100), (101), and (102) reflections, confirming the polycrystalline structure. However, the FFT over the smaller region (#2), which is more of a single-crystal-like domain with the lattice spacing of ∼3.05 Å (corresponding to (100) reflection) exhibits hexagonal crystal symmetry (Fig. 10d (inset)) rather than polycrystalline rings.

**Fig. 11:**
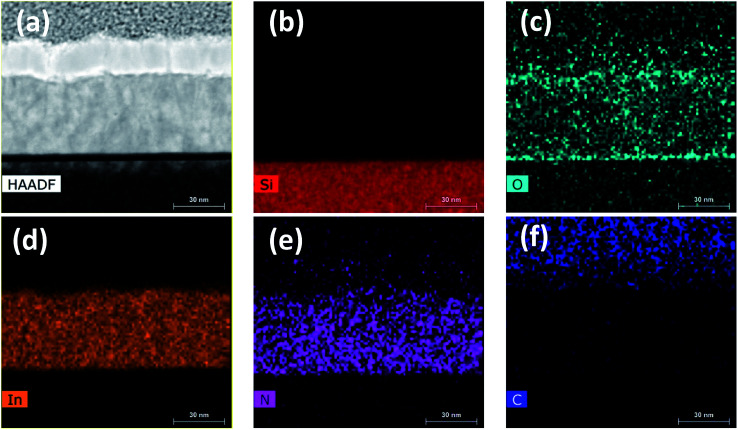
(a) HR-STEM micrograph of 600-cycle InN film grown at 240 °C with 100 W rf-power using Ar/N_2_ plasma composition. (b)–(f) EDX elemental mapping over the cross-section of the sample showing silicon (Si), oxygen (O), indium (In), nitrogen (N), and carbon (C) content of the film.

Fig. 11 shows the cross-sectional HR-S/TEM image and EDX elemental maps of In, N, O, C, and Si for the InN sample grown at 240 °C. Si, In, N, and C show strong contrasts over the scanned region on their respective color-mapped micrographs. Presence of significant carbon on the protective capping layer, most probably stems from the organic ligands of Pt precursor used in the FIB system, where it dramatically fades out on the scanned region over the bulk of InN film (Fig. 11f). Also, the presence of O almost over the whole scanned area of the sample (Fig. 11c) is due to the atmospheric exposure of the sample. Also, the rather weak N signal extending into the AuPd layer (Fig. 11e) falls within the EDX background noise and thus can be ignored. However, In and N signals with much stronger presence appear to be more homogeneous within the bulk of InN, Fig. 11d and e. Therefore, EDX elemental mapping also agrees well with the XPS extracted elemental composition for the 240 °C grown InN sample.

## Conclusions

In this work, we have explored and attempted to understand the role of varying nitrogen plasmas on the low-temperature self-limiting growth of InN films. Real-time *in situ* ellipsometry was effectively utilized to monitor and detect instantaneous film thickness variations during ALD cycles as a function of varying plasma conditions without interfering the growth process. GIXRD analysis showed that single-phase polycrystalline h-InN was achieved with both Ar/N_2_ and N_2_-only plasmas; whereas, when hydrogen was admixed with Ar/N_2_ plasma chemistry, led to a drastic microstructural change resulting in crystalline oxide films (c-In_2_O_3_). XPS measurements revealed that near-ideal stoichiometric InN was produced with Ar/N_2_ and N_2_-only plasma compositions. On the other hand, highly nitrogen-deficient InO_*x*_N_*y*_ films with ∼44% and ∼5% oxygen and nitrogen incorporation, respectively, were formed under Ar/N_2_/H_2_-plasma. This dramatic change in film composition and structure was attributed to hydrogen radicals mainly promoting hydroxyl (–OH) formation on the pre-adsorbed TMI surface groups during the plasma exposure half-cycles. The main source of oxygen in this process was assigned to residual water-vapor being trapped within multilayer film stacks on the reactor chamber. Independent of the plasma chemistries employed, all of the films exhibited below-detection limit carbon content within the bulk. InN films grown with 100 W Ar/N_2_ plasma within 200–240 °C temperature range revealed hexagonal polycrystalline wurtzite structure with slightly improved peak intensity at 240 °C, while showing an amorphous character at 160 °C, mainly due to insufficient surface energy needed for crystallization. Varying the rf-power of the hollow-cathode plasma source to 50 and 150 W resulted in similar structural amorphous character. While in the lower rf-power regime, the surface energy is not sufficient to trigger crystallization, on the other hand, the higher rf-power leads to plasma-induced crystal damage, both resulting in amorphous or weakly-crystalline InN films. The InN sample grown at optimal plasma conditions at 240 °C, exhibited clear polycrystalline layer character with ∼20 nm average-sized single-crystal InN domains showing hexagonal symmetry.

## Conflicts of interest

The authors declare no conflicts of interest.

## Supplementary Material

RA-010-D0RA04567E-s001
