# Supplementary material for: Elucidating the role of nitrogen plasma composition in the low-temperature self-limiting growth of indium nitride thin films
Source: RSC Adv. 2020 Jul 21;10(46):27357–68. doi: 10.1039/d0ra04567e (PMC9055617; doi:10.1039/d0ra04567e)
Supplement: RA-010-D0RA04567E-s001 [file RA-010-D0RA04567E-s001.pdf]

Electronic Supplementary Material (ESI) for RSC Advances.
This journal is © The Royal Society of Chemistry 2020

# Elucidating the role of hydrogen radicals in the low-temperature self-limiting surface reactions between trimethylindium and nitrogen plasmas

*Supplemental Data*

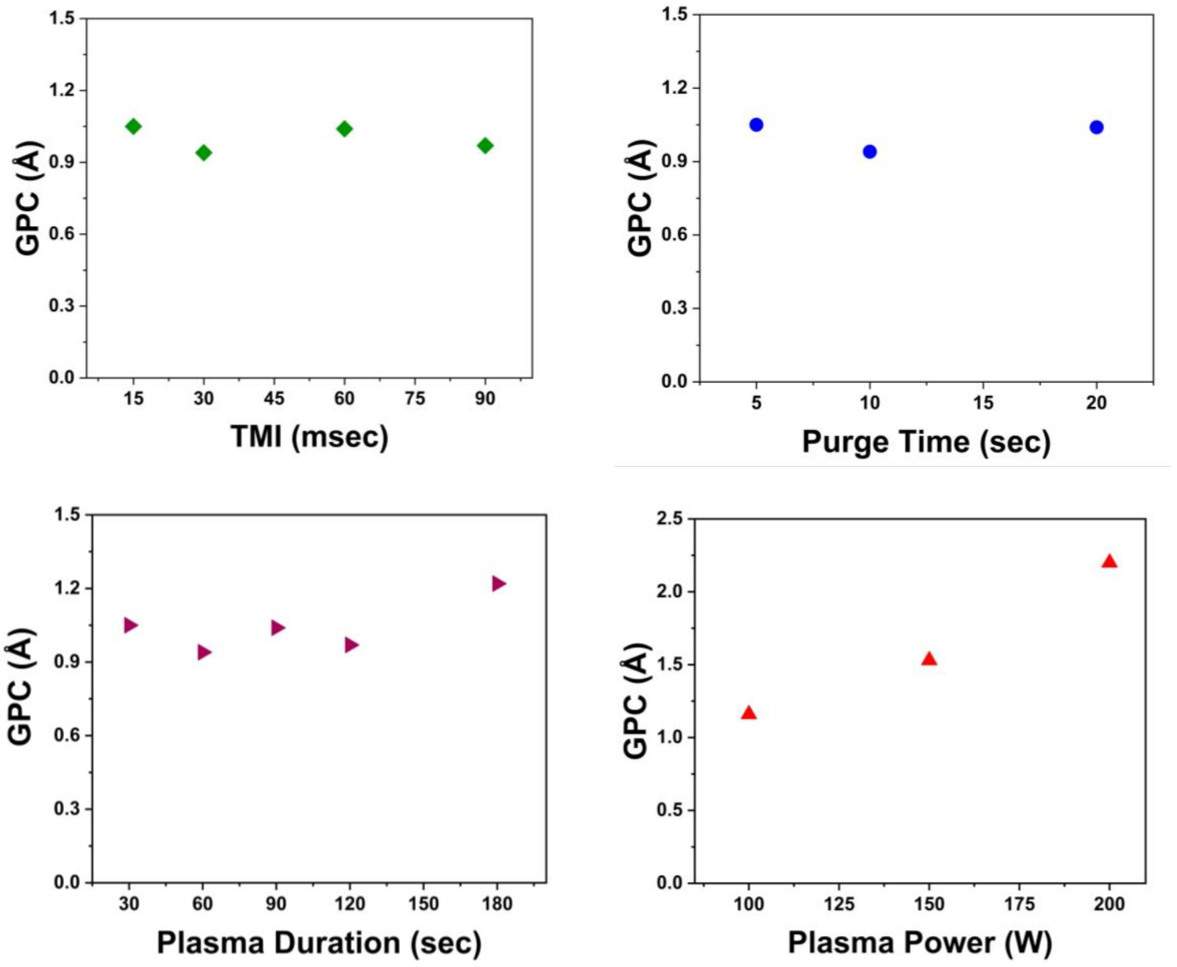


**FIG. S1.** Saturation curves for InN film growth at 200 °C and 50/50 sccm of Ar/$N_2$ plasma composition at: (a) varying TMI dose with 100 W, 60 sec exposure time. (b) different purge times with 30 msec TMI dose, 100 W, 60 sec exposure time. (c) different plasma durations, 100 W, 30 msec TMI, and 10 sec purge periods. (d) varying rf-plasma powers, 60 sec plasma time, 30 msec TMI pulse, and 10 sec purge times each.

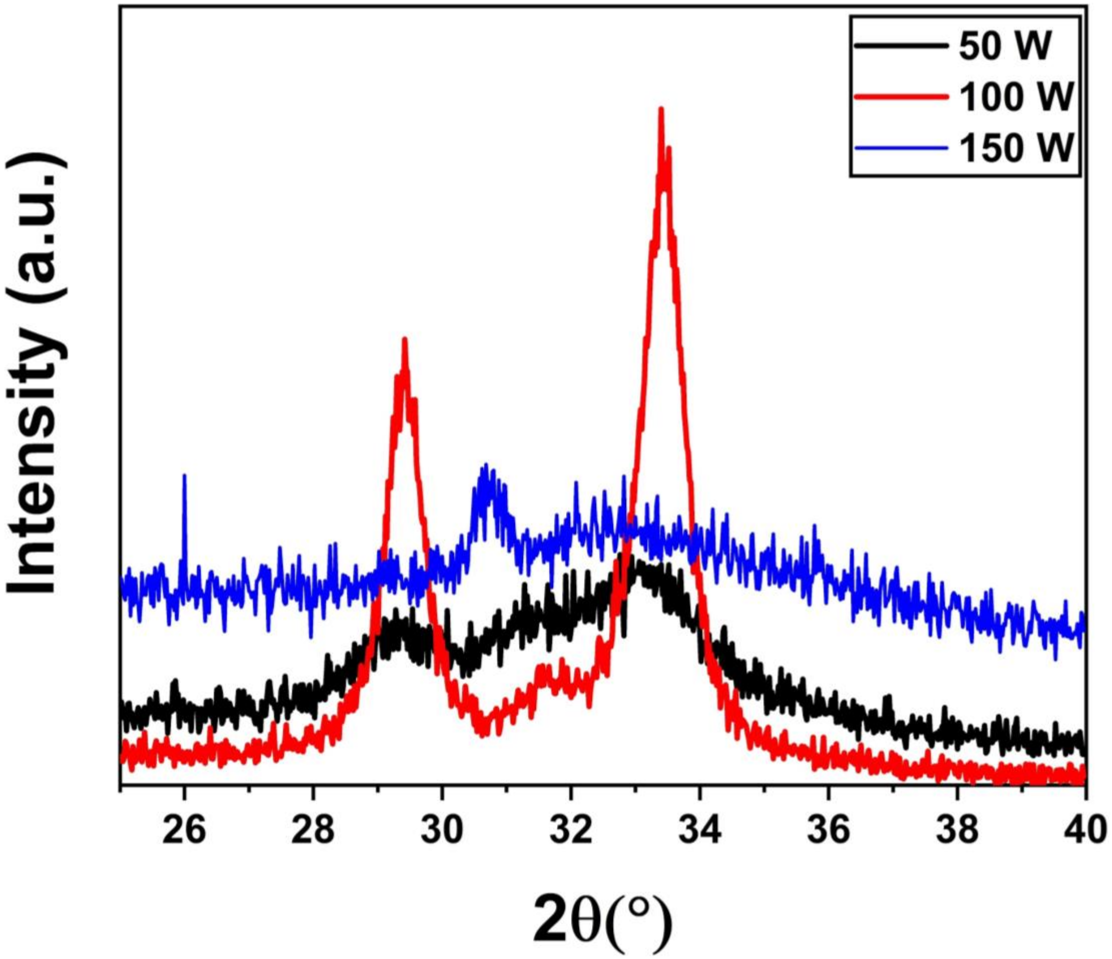


**FIG. S2.** GIXRD spectra for the 600-cycles grown InN sample using Ar/$N_2$ plasma chemistry at 200 °C, with varying rf-plasma powers.

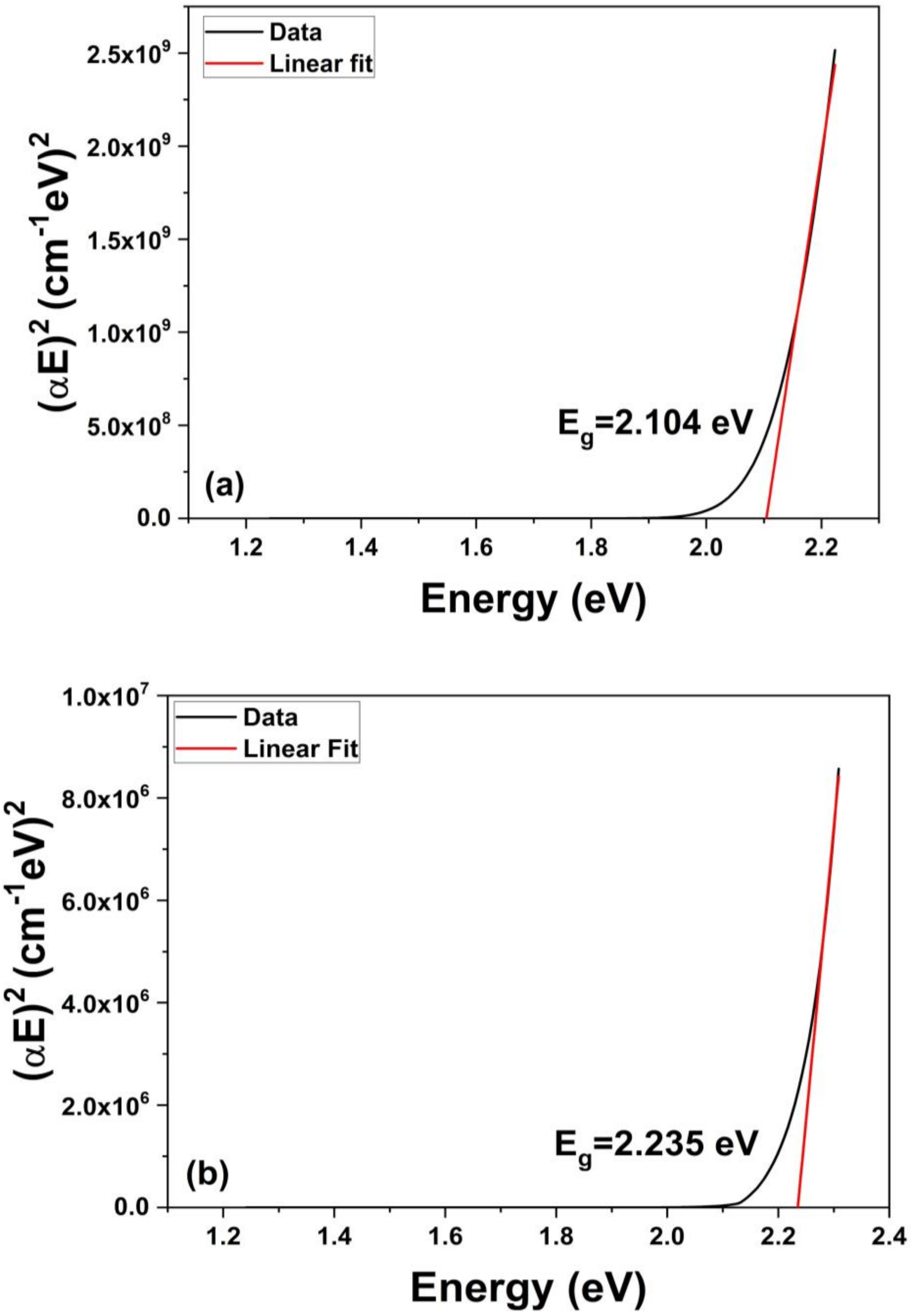


**FIG. S3.** (a) Spectral absorption behavior of InN film deposited at 200°C, along with the extracted absorption band edge fit. (b) Spectral absorption behavior of InN film deposited at 160°C, along with the extracted absorption band edge fit.
